# Gallbladder Volvulus: A Rare Cause of Acute Abdomen in the Elderly

**DOI:** 10.7759/cureus.97198

**Published:** 2025-11-19

**Authors:** Daiki Sato, Guillaume Aeby, Birgit M Helmchen, Reint Burger

**Affiliations:** 1 General Surgery, Hospital Männedorf, Männedorf, CHE; 2 Pathology and Molecular Pathology, University Hospital of Zürich, Zürich, CHE

**Keywords:** acute acalculous cholecystitis (aac), emergency diagnostics, gallbladder volvulus, lap converted to open cholecystectomy, surgical acute abdomen

## Abstract

Gallbladder volvulus (GV) is a rare but potentially life-threatening condition characterized by the torsion of the gallbladder around its vascular pedicle. It predominantly affects elderly females and is often underdiagnosed. We report the case of an 84-year-old female patient with a history of deep vein thrombosis on rivaroxaban, presenting with acute upper abdominal pain. On admission, the patient was hemodynamically stable but in a poor general condition. Physical examination revealed tenderness in the right upper quadrant without guarding. Laboratory tests showed significantly elevated inflammatory markers and electrolyte disturbances. Abdominal CT demonstrated a distended gallbladder located in the mid-abdomen with a thickened wall and mild ascites. No radiopaque gallstones were detected. Empirical intravenous antibiotic therapy with amoxicillin/clavulanic acid was initiated. Due to persistent symptoms and worsening inflammatory parameters, a laparoscopic cholecystectomy was performed after discontinuation of anticoagulation therapy. Intraoperatively, a 180° torsion of the gallbladder around the infundibulum was identified. Due to a lack of adequate visualization, conversion to an open cholecystectomy was performed. Histopathological examination confirmed transmural ischemia, hemorrhage, and purulent inflammation without malignancy. The postoperative course was uneventful, and the patient was discharged in good general condition on the 11th postoperative day. GV should be considered in elderly patients presenting with atypical acute cholecystitis symptoms. Prompt surgical intervention is essential to prevent severe complications such as gangrene and perforation.

## Introduction

Acute cholecystitis is among the most frequent diagnoses in emergency care units, causing acute abdominal pain [1], typically resulting from cystic duct obstruction by gallstones. However, in rare cases, inflammation of the gallbladder can occur secondary to gallbladder volvulus (GV), a torsion of the gallbladder around its mesentery, leading to vascular compromise and ischemia. Typical symptoms resemble acute calculous cholecystitis [2], making preoperative diagnosis difficult. Delay in recognition can result in gangrene, perforation, and sepsis, contributing to significant morbidity and mortality [3]. Given its rarity and diagnostic difficulty, reporting new cases of GV remains important to raise clinical awareness and emphasize distinguishing radiologic and intraoperative features. This report aims to highlight the diagnostic challenges, perioperative considerations, and management principles in an elderly patient presenting with GV masquerading as acute cholecystitis.

## Case presentation

Medical history and clinical status

In November 2022, an 84-year-old female patient was referred from a nursing home due to reported hematemesis and upper abdominal pain persisting for two days. The patient had regular bowel movements. Her symptoms had not improved despite high-dose pantoprazole administration. Due to mild dementia, additional subjective complaints could not be obtained. She was on medication, including rivaroxaban 20 mg orally, due to a history of deep vein thrombosis. She had no previous abdominal surgeries. Upon admission, the patient was cardiopulmonary stable and afebrile but exhibited poor general condition. Physical examination revealed sparse bowel sounds on auscultation. Palpation elicited marked tenderness in the right upper abdomen without signs of peritonitis. Hematemesis was not observed during admission.

Further diagnostics

**Figure 1:**
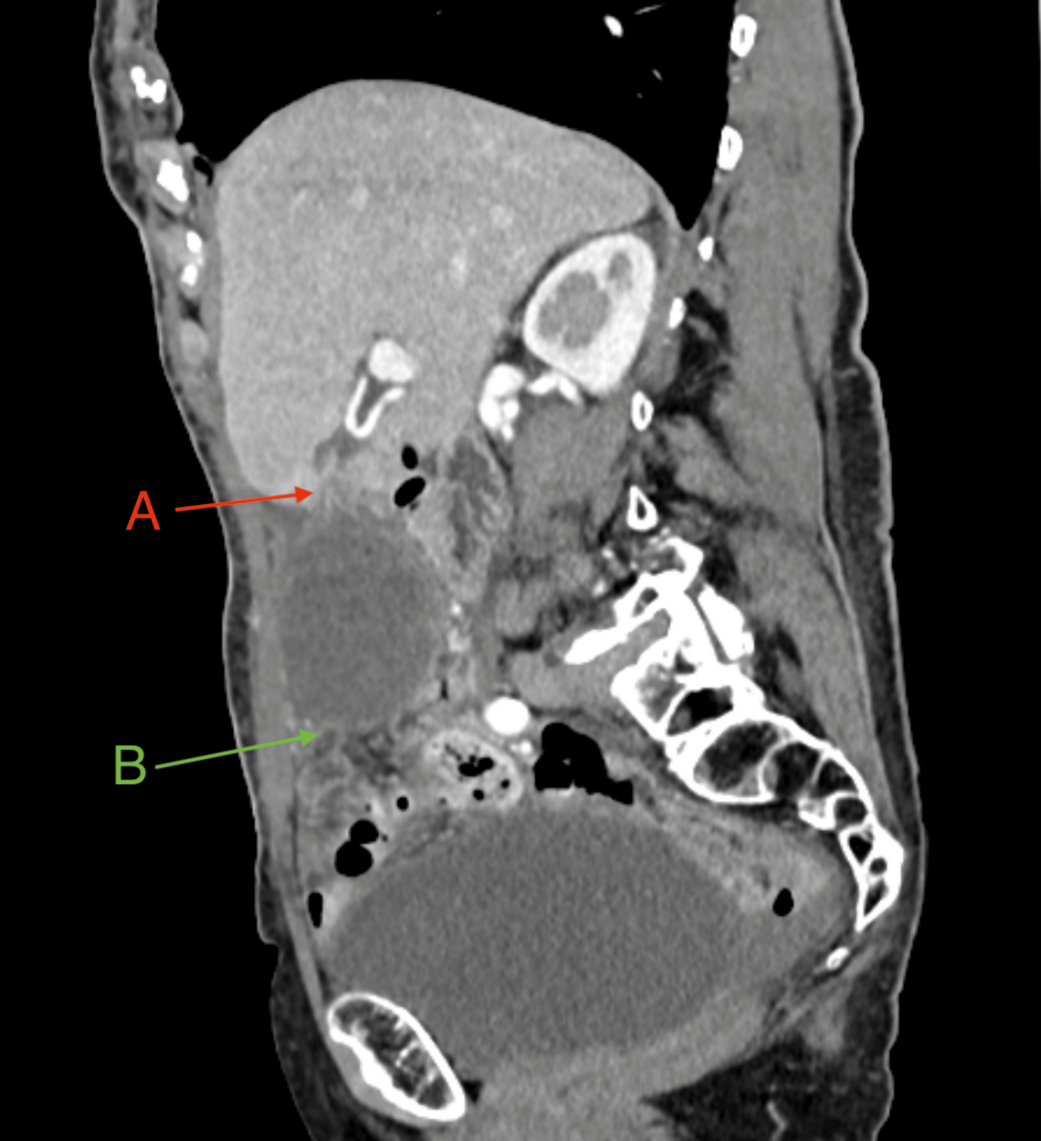
CT abdomen, sagittal plane (early arterial phase). (A) Filling defect of the cystic artery (*arteria cystica*). (B) Gallbladder hydrops with pericystic fluid.

**Figure 2:**
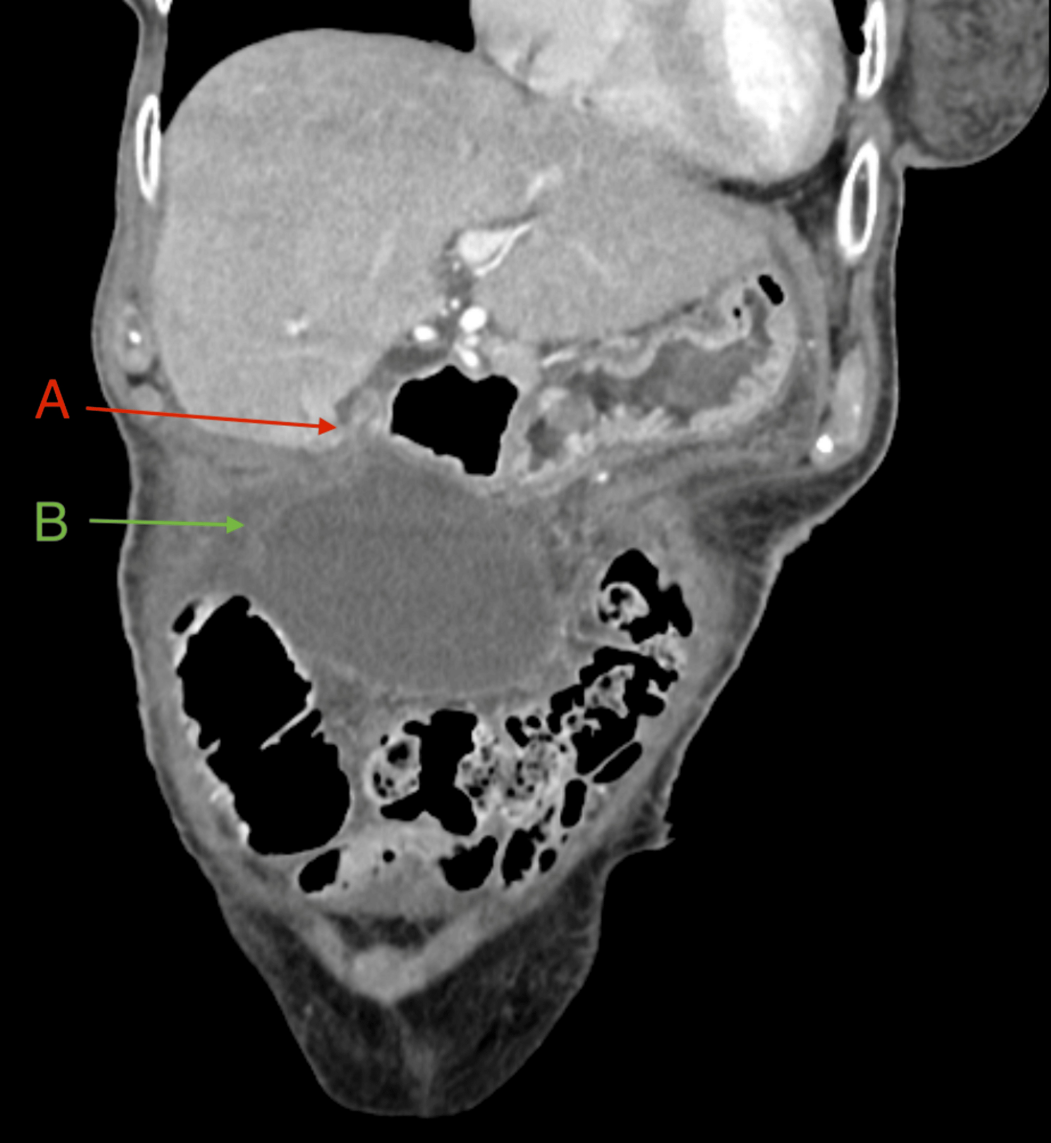
CT abdomen, coronal plane (early arterial phase). (A) Filling defect of the cystic artery (*arteria cystica*). (B) Gallbladder hydrops with pericystic fluid.

Laboratory tests revealed significantly elevated inflammatory markers (CRP 323 mg/L; reference <5mg/L, leucocytosis 17,000/µL; reference 4-10,000/µL) along with pronounced hyponatremia (serum sodium 125 mmol/L; reference 136-145mmol/L). Additionally, slightly elevated cholestasis (serum bilirubin 22 µmol/L; reference <17µmol/L) and liver enzyme parameters were noted (aspartate aminotransferase (AST), also known as glutamic-oxaloacetic transaminase (GOT) 37 U/L; reference 10-50U/L). The hemoglobin levels were normal upon arrival (hemoglobin 136g/L; reference 117-153 g/L, hematocrit 0.385; reference 0.350-0.460). Initial ultrasonography in the emergency department showed an unclear cystic mass without hyperechoic structures, extending from the right middle to the lower abdomen. An abdominal CT (arterial, venous, and delayed phases) was performed, demonstrating pronounced gallbladder hydrops with displacement of the gallbladder into the mid-abdomen and distinct wall thickening (Figures 1-2). The common bile duct was borderline dilated (8 mm), and the cystic duct showed increased contrast uptake. No radiopaque stones were identified. Mild ascites was present in all four abdominal quadrants. No signs of perforation, gangrene, or fistulation were noted.

Treatment and clinical course

**Figure 3:**
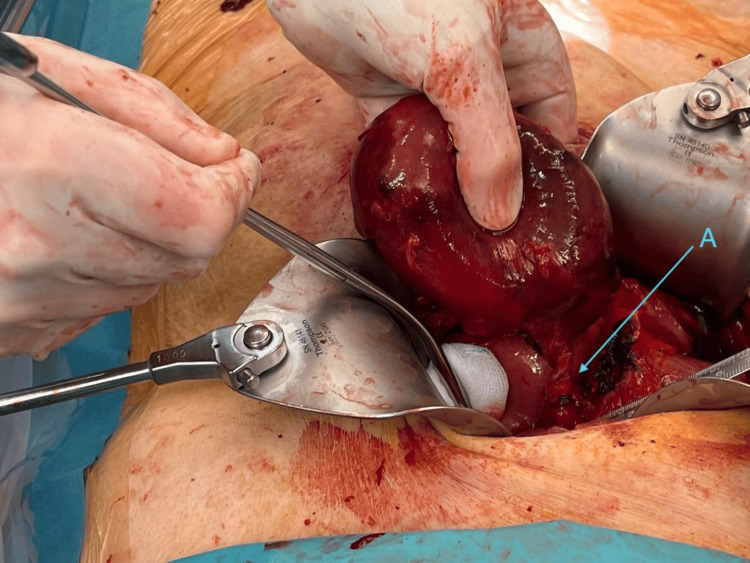
Intraoperative documentation. (A) Gallbladder with visible torsion around the cystic artery (*arteria cystica*) and cystic duct (*ductus cysticus*).

**Figure 4:**
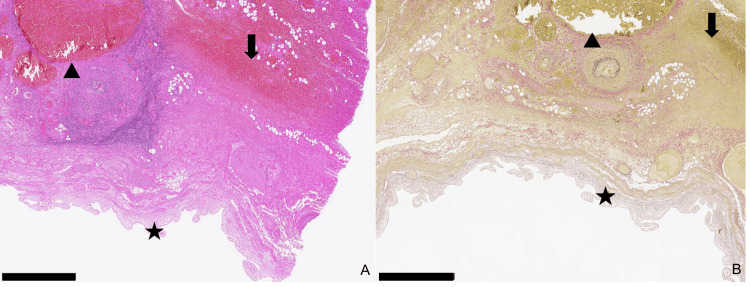
Histopathological sections of the gallbladder. (A) Hematoxylin and eosin stain, 2.5×. (B): Elastica van Gieson stain, 2.5×. Scale bar: 1 mm; Star: Necrotic, degenerating mucosa; Triangle: Blood-congested vessels; Arrow: Interstitial hemorrhage.

Given her ongoing rivaroxaban 20 mg therapy and stable cardiopulmonary status, the patient was initially admitted for empirical intravenous antibiotic therapy with amoxicillin/clavulanic acid. Rivaroxaban therapy was discontinued. Clinically, the patient remained cardiopulmonary stable; however, due to persistent pain and worsening inflammatory markers (CRP increased to 365 mg/L; reference <5 mg/L), laparoscopic cholecystectomy was performed two days after discontinuing anticoagulation. Intraoperative laparoscopy confirmed significant gallbladder hydrops extending to the infraumbilical region, with additional inflammatory adhesions between the greater omentum and the abdominal wall. Due to poor visualization, conversion to open surgery via a right subcostal laparotomy (Kocher) was necessary. The gallbladder was successfully removed, revealing a 180-degree counterclockwise torsion at the infundibulum level (Figure 3). The cholecystectomy proceeded without complications. Due to markedly elevated inflammatory markers, antibiotic therapy was continued for seven days postoperatively. Histopathology showed transmural ischemia, hemorrhages, and extensive purulent inflammation without malignancy (Figure 4). Due to the reported hematemesis, endoscopic examination excluded ulceration but incidentally revealed grade III reflux esophagitis (Savary-Miller classification) [4]. The patient was discharged in good general condition back to the nursing home on the 11th postoperative day. A clinical follow-up was conducted six weeks postoperatively. According to the nursing home staff and family members, the patient showed no signs of gastrointestinal or pulmonary complications and remained in adequate pain control with paracetamol and metamizole. No wound infections or thromboembolic events were noted.

## Discussion

GV is a rare medical condition in which the gallbladder twists around its longitudinal axis, including the cystic artery (*arteria cystica*) and cystic duct (*ductus cysticus*) [3]. Since its initial description in 1898 up to 2020, a total of 477 cases have been reported, of which only 80 cases (17%) were diagnosed preoperatively [5,6]. The pathophysiology of GV is multifactorial, including liver atrophy, weight loss, loss of visceral fat, and anatomical variations related to the liver, potentially causing elongation of the gallbladder mesentery and subsequent torsion [7]. Epidemiologically, GV exhibits a bimodal distribution with a small peak in pediatric patients and a larger peak in those over 70 years. In elderly populations, women are affected more than men at a ratio of roughly 5:1, in contrast to the male predominance in pediatric cases [6].

Clinically and on laboratory tests, GV is difficult to distinguish from other causes of acute cholecystitis. Ultrasonography may detect an atypical gallbladder position, and Doppler ultrasound may assess blood flow [8]. CT and magnetic resonance cholangiopancreatography (MRCP) can identify pathognomonic signs of GV, including a distended gallbladder, pericholecystic fluid, atypical position of the gallbladder, lack of contrast enhancement, and rapid tapering of the cystic duct and artery [9-11]. Our patient illustrates many of these clinical and radiographic features. She was an elderly female presenting with upper abdominal pain and inflammatory markers. Although typical CT signs were present, the diagnosis of GV was only established intraoperatively, as in most reported cases [6]. This reflects the diagnostic challenges encountered in GV despite preoperative CT scans, possibly due to the rarity of the condition. Moreover, our patient’s history of anticoagulation therapy required careful perioperative management and contributed to delaying surgery, a factor not frequently highlighted in prior reports. In addition, reported hematemesis initially suggested an upper gastrointestinal source, potentially diverting attention away from the biliary tract. These case-specific features underscore how comorbidities and atypical symptoms can complicate the timely diagnosis of GV.

The treatment of choice for GV remains emergency cholecystectomy, either laparoscopic or open. Since the first laparoscopic cholecystectomy for GV in 1995 [12], minimally invasive procedures have been increasingly employed. However, systematic comparative studies of the two methods are currently lacking. Due to the abnormal anatomy, the common bile duct may be positioned anteriorly, increasing its vulnerability to iatrogenic injuries; therefore, extreme caution during dissection is advised. Critical operative steps include decompression by puncture, detorsion, and achieving the "Critical View of Safety" [13]. In our case, laparoscopic exploration was attempted but required conversion due to poor visualization and risk of iatrogenic injury. Conservative treatment with broad-spectrum antibiotics is associated with a poor prognosis. Percutaneous cholecystostomy has been unsuccessful, as it does not correct the underlying anatomical anomaly and carries the risk of delaying definitive surgical treatment. Delaying surgery for more than two days after symptom onset is associated with poor prognosis and increased mortality. Untreated GV may progress to ischemia, gallbladder necrosis, perforation, and ultimately sepsis. Overall morbidity and mortality are 16% and 6%, respectively [6].

This case has several limitations. First, the preoperative imaging did not conclusively establish the diagnosis, highlighting the difficulty of radiologic recognition of GV. Second, we were unable to include complete ultrasonographic media, which would have strengthened the documentation. Finally, as a single case report, the findings cannot be generalized but rather serve to raise awareness of GV as a differential diagnosis in elderly patients with atypical acute cholecystitis.

## Conclusions

GV is a rare but serious cause of acute abdomen, particularly in elderly women, and should be considered when imaging reveals gallbladder displacement or hydrops without gallstones. This case emphasizes three key learning points: first, anticoagulation and comorbidities may complicate surgical timing but should not delay intervention once GV is suspected. Second, preoperative diagnosis remains challenging, requiring a high index of suspicion in atypical presentation of acute cholecystitis; and third, timely cholecystectomy is essential to prevent ischemia, necrosis, and perforation, directly impacting morbidity and mortality. Greater awareness of this entity may facilitate earlier recognition and improve outcomes.
